# Semi-Supervised Minimum Error Entropy Principle with Distributed Method

**DOI:** 10.3390/e20120968

**Published:** 2018-12-14

**Authors:** Baobin Wang, Ting Hu

**Affiliations:** 1 School of Mathematics and Statistics, South-Central University for Nationalities, Wuhan 430074, China; 2 School of Mathematics and Statistics, Wuhan University, Wuhan 430072, China

**Keywords:** information theoretical learning, distributed method, MEE algorithm, semi-supervised approach, gradient descent, reproducing kernel Hilbert spaces

## Abstract

The minimum error entropy principle (MEE) is an alternative of the classical least squares for its robustness to non-Gaussian noise. This paper studies the gradient descent algorithm for MEE with a semi-supervised approach and distributed method, and shows that using the additional information of unlabeled data can enhance the learning ability of the distributed MEE algorithm. Our result proves that the mean squared error of the distributed gradient descent MEE algorithm can be minimax optimal for regression if the number of local machines increases polynomially as the total datasize.

## 1. Introduction

The minimum error entropy (MEE) principle is an important criterion proposed in information theoretical learning (ITL) [1] and was firstly addressed for adaptive system training by Erdogmus and Principe [2]. It has been applied to blind source separation, maximally informative subspace projections, clustering, feature selection, blind deconvolution, minimum cross-entropy for model selection, and some other topics [3,4,5,6,7,8]. Taking entropy as a measure of the error, the MEE principle can extract the information contained in data fully and produce robustness to outliers in the implementation of algorithms.

Let $X\in R^{n}$ be an explanatory variable with values taken in a compact metric space (X,d),*Y* be a real response variable with Y∈Y⊂R, and g:X→Y be a prediction function. For a given set of labeled examples $D={\left\{\left(x_{i},y_{i}\right)\right\}}_{i=1}^{N}\subset X\times Y$ (*N* denotes the sample size) and a windowing function $G:R\to R_{+},$ the MEE principle is to find a minimizer of the empirical quadratic entropy:

$$\begin{array}{c} \hat{H}\left(g\right)=-\log\left\{\frac{h^{2}}{N^{2}}\sum_{\begin{array}{c} (x_{i},y_{i})\in D\\ (x_{j},y_{j})\in D \end{array}}G\left(\frac{{\left[\left(y_{i}-g\left(x_{i}\right)\right)-\left(y_{j}-g\left(x_{j}\right)\right)\right]}^{2}}{h^{2}}\right)\right\}, \end{array}$$

where h>0 is the scaling parameter. Its goal is to solve the problem $y=g_{\rho }\left(x\right)+\varepsilon$, where ε is the noise and $g_{\rho }\left(x\right)$ is the target function. Taking a function $f\left(x_{i},x_{j}\right):=g\left(x_{i}\right)-g\left(x_{j}\right),$ MEE belongs to pairwise learning problems, which involves with the intersections of example pairs. Since logarithmic function is monotonic, we only consider the empirical information error of MEE:

(1) $$\begin{array}{c} R\left(f\right)=-\frac{h^{2}}{N^{2}}\sum_{\begin{array}{c} (x_{i},y_{i})\in D\\ (x_{j},y_{j})\in D \end{array}}G\left(\frac{{\left[y_{i}-y_{j}-f\left(x_{i},x_{j}\right)\right]}^{2}}{h^{2}}\right), \end{array}$$

in the optimization process. Borrowing the idea from Reference [9], we introduced the Mercer kernel $K\left(\cdot ,\cdot \right):X^{2}\times X^{2}\to R,\left(X^{2}:=X\times X\right)$ and employed the reproducing kernel Hilbert space (RKHS) $H_{K}$ as our hypothesis space. With K, $H_{K}$ is defined as the linear span of the functions set $\{K_{\left(x,u\right)}:=K\left((x,u),(\cdot ,\cdot )\right),\forall \left(x,u\right)\in X^{2}\},$ which is equipped with the inner product ${\langle \cdot ,\cdot \rangle }_{K}$ and the reproducing property ${\langle K_{\left(x,u\right)},K_{\left(x^{\prime },u^{\prime }\right)}\rangle }_{K}=K\left(\left(x,u\right),\left(x^{\prime },u^{\prime }\right)\right),\forall \left(x,u\right),\left(x^{\prime },u^{\prime }\right)\in X^{2}.$ For the *G* nonconvex, we usually solve Equation (1) using the kernel-based gradient descent method as follows. It starts with $f_{1,D}=0$ and is updated by:

(2) $$\begin{array}{c} f_{t+1,D}=f_{t,D}-\eta \times \nabla R\left(f_{t,D}\right), \end{array}$$

in the *t*-th step, where η>0 is a step size, ∇ is the gradient operator and:

$$\nabla R\left(f_{t,D}\right)=-\frac{1}{N^{2}}\sum_{\begin{array}{c} (x_{i},y_{i})\in D\\ (x_{j},y_{j})\in D \end{array}}G^{\prime }\left(\frac{{\left[y_{i}-y_{j}-f_{t,D}\left(x_{i},x_{j}\right)\right]}^{2}}{h^{2}}\right)\left[f_{t,D}\left(x_{i},x_{j}\right)-y_{i}+y_{j}\right]K_{\left(x_{i},x_{j}\right)},$$

as we know that the example pairs will grow quadratically with the increasing example size *N*, which will bring the computational burden in the MEE implementation. Thus, it is necessary to reduce the algorithmic complexity by the distributed method based on a divide-and-conquer strategy [10]. Semi-supervised learning (SSL) [11] has attracted extensive attention as an emerging field in machine learning research and data mining. Actually, in many practical problems, few data are given, but a large number of unlabeled data are available, since labeling data requires a lot of time, effort or money. In this paper, we study a distributed MEE algorithm in the framework of SSL and show that the learning ability of the MEE algorithm can be enhanced by the distributed method and the combination of labeled data with unlabeled data.

There are mainly three contributions in this paper. The first one is that we derive the explicit learning rate of the gradient descent method for distributed MEE in the context of SSL, which is comparable to the minimax optimal rate of the least squares in regression. This implies that the MEE algorithm can be an alternative of the least squares in SSL in the sense that both of them have the same prediction power. The second one is that we provide the theoretical upper bound for the number of local machines guaranteeing the optimal rate in the distributed computation. The last one is that we extend the range of the target function allowed in the distributed MEE algorithm.

In Table 1, we summarize some notations used in this paper.

## 2. Algorithms and Main Results

We considered MEE for the regression problem. To allow noise in sampling processes, we assumed that a Borel measure ρ(·,·) is defined on the product space X×Y. Let ρ(y|x) be the conditional distribution of y∈Y for any given x∈X, and $\rho_{X}\left(\cdot \right)$ the marginal distribution on X. For the semi-supervised MEE algorithm, our goal was to estimate the regression function $g_{\rho }\left(x\right)=\int_{Y}yd\rho \left(y|x\right),x\in X$, from labeled examples $D={\left\{\left(x_{i},y_{i}\right)\right\}}_{i=1}^{N}$ and unlabeled examples $D^{*}={\left\{x_{j}\right\}}_{j=1}^{S}$ drawn from the distribution ρ and $\rho_{X}$, respectively.

Based on the divide-and-conquer strategy, both *D* and $D^{*}$ are partitioned equally into *m* subsets, $D=\sum_{l=1}^{m}⋃D_{l}$ and $D^{*}=\sum_{l=1}^{m}⋃D_{l}^{*}.$ Here, we denote the size of subsets $|D_{l}|=n$ and $|D_{l}^{*}|=s$, 1≤l≤m, i.e., N=mn,S=ms. We construct a new dataset $\tilde{D}=\sum_{l=1}^{m}⋃{\tilde{D}}_{l}$ by:

$$\begin{array}{c} {\tilde{D}}_{l}=D_{l}\cup D_{l}^{*}={\left\{\left(x_{k},y_{k}\right)\right\}}_{k=1}^{n+s}, \end{array}$$

where:

$$x_{k}=\left\{\begin{array}{cc} x_{k}, & \mathrm{if}\;\left(x_{k},y_{k}\right)\in D_{l},\\ x_{k}, & \mathrm{if}\;x_{k}\in D_{l}^{*}, \end{array}\right.\;\mathrm{and}\;y_{k}=\left\{\begin{array}{cc} \frac{n+s}{n}y_{k}, & \mathrm{if}\;\left(x_{k},y_{k}\right)\in D_{l},\\ 0, & \mathrm{if}\;x_{k}\in D_{l}^{*}. \end{array}\right.$$

Based on the gradient descent algorithm (Equation (2)), we can get a set of local estimators $\left\{f_{t,{\tilde{D}}_{l}}\right\}$ for each subset ${\tilde{D}}_{l},1\leq l\leq m.$ Then, the global estimator averaging over these local estimators is given by:

(3) $$\begin{array}{c} {\bar{f}}_{t,\tilde{D}}=\frac{1}{m}\sum_{l=1}^{m}f_{t,{\tilde{D}}_{l}}. \end{array}$$

In the pairwise setting, our target function $f_{\rho }\left(x,x^{\prime }\right)=g_{\rho }\left(x\right)-g_{\rho }\left(x^{\prime }\right),x,x^{\prime }\in X,$ which is the difference of the regression function $g_{\rho }.$ Denote by $L_{\rho_{X^{2}}}^{2}$ the space of square integrable functions on the product space $X^{2}$:

$$\begin{array}{c} L_{\rho_{X^{2}}}^{2}:=\left\{f:X^{2}\to R:{∥f∥}_{L^{2}}={\left(\int \int_{X^{2}}{\left|f\left(x,x^{\prime }\right)\right|}^{2}d\rho_{X}\left(x\right)d\rho_{X}\left(x^{\prime }\right)\right)}^{\frac{1}{2}}<\infty \right\}. \end{array}$$

The goodness of ${\bar{f}}_{t,\tilde{D}}$ is usually measured by the mean squared error $∥{\bar{f}}_{t,\tilde{D}}-f_{\rho }{∥}_{L^{2}}^{2}.$

Throughout the paper, we assumed that $\underset{\left(x,x^{\prime }\right)\in X^{2}}{\sup}\sqrt{K(\left(x,x^{\prime }\right),\left(x,x^{\prime }\right))}\leq 1$ and for some constant M>0, |y|≤M almost surely. Without generality, windowing function *G* is assumed to be differentiable and satisfies $G^{\prime }\left(0\right)=-1,$ $G^{\prime }\left(u\right)<0$ for u>0, $C_{G}:=\sup_{u\in (0,\infty )}\left|G^{\prime }\left(u\right)\right|<\infty$ and there exists some *p* such that $c_{p}>0$ and:

(4) $$\begin{array}{c} |G^{\prime }\left(u\right)-G^{\prime }\left(0\right)|\leq c_{p}{\left|u\right|}^{p},\;\forall u>0. \end{array}$$

It is easy to check that the Gaussian kernel G(u)=exp{-u} satisfies the assumptions above with p=1.

Before we present our main results, define an integral operator $L_{K}:L_{\rho_{X^{2}}}^{2}⟶L_{\rho_{X^{2}}}^{2}$ associated with the kernel *K* by:

$$\begin{array}{c} L_{K}\left(f\right):=\int_{X}\int_{X}f\left(x,x^{\prime }\right)K_{\left(x,x^{\prime }\right)}d\rho_{X}\left(x\right)d\rho_{X}\left(x^{\prime }\right),\;\forall f\in L_{\rho_{X^{2}}}^{2}. \end{array}$$

Our error analysis for the distributed MEE algorithm (Equation (3)) is stated in terms of the following regularity condition:

(5) $$\begin{array}{c} f_{\rho }=L_{K}^{r}\left(\phi \right)\;for\;some\;r>0,\;\phi \in L_{\rho_{X^{2}}}^{2}, \end{array}$$

where $L_{K}^{r}$ denotes the *r*-th power of $L_{K}$ on $L_{\rho_{X^{2}}}^{2}$ and is well defined, since the operator $L_{K}$ is positive and compact with the Mercer kernel K. We use the effective dimension [12,13] N(λ) to measure the complexity of $H_{K}$ with respect to $\rho_{X},$ which is defined to be the trace of the operator ${\left(\lambda I+L_{K}\right)}^{-1}L_{K}$ as:

$$\begin{array}{c} N\left(\lambda \right)=Tr({\left(\lambda I+L_{K}\right)}^{-1}L_{K}),\lambda >0. \end{array}$$

To obtain optimal learning rates, we need to quantify N(λ) of $H_{K}$. A suitable assumption is: that

(6) $$\begin{array}{c} N\left(\lambda \right)\leq C_{0}\lambda^{-\beta },for\;some\;C_{0}>0\;and\;0<\beta \leq 1. \end{array}$$

**Remark** **1.** *When β=1, Equation (6) always holds with $C_{0}=Tr\left(L_{K}\right).$ For 0<β<1, when $H_{K}$ is a Sobolev space $W^{\alpha }\left(X\right)$ on $X\subset R^{d}$ with all derivative of order up to $\alpha >\frac{d}{2},$ then Equation (6) is satisfied with $\beta =\frac{d}{2\alpha }$ [14]. Moreover, if the eigenvalues ${\left\{\gamma_{i}\right\}}_{i=1}^{\infty }$ of the operator $L_{K}$ decays as $\gamma_{i}=O\left(i^{-b}\right)$ for some b>1, then $N\left(\lambda \right)=O\left(\lambda^{-\frac{1}{b}}\right).$ The eigenvalues assumption is typical in the analysis of the performances of kernel methods estimators and recently used in References [13,15,16] to establish the optimal learning rate in the least square problems.*

The following theorem shows that the distributed gradient descent algorithm (Equation (3)) can achieve the optimal rate by providing the iteration time *T* and the maximal number of local machines, whose proof can be found in Section 3.

**Theorem 1.** **(Main Result)** *Assume Equations (5) and (6) hold for* $r+\beta \geq \frac{1}{2}.$ *Let the iteration time* $T={\left\lceil N/4\right\rceil }^{\frac{1}{2r+\beta }}$ *and* $S+N\geq N^{\frac{\beta +1}{2r+\beta }}$ *:*

(7) $$\begin{array}{c} m<\frac{\min\{{\left(N+S\right)}^{\frac{1}{2}}N^{-\frac{\beta +1}{4r+2\beta }},{\left(N+S\right)}^{\frac{1}{3}}N^{-\frac{2-2r-\beta }{6r+3\beta }}\}}{\log^{6}N}, \end{array}$$

*then for any 0<δ<1, with confidence at least 1-δ:*

(8) $$\begin{array}{c} ∥{\bar{f}}_{T+1,\tilde{D}}-f_{\rho }{∥}_{L^{2}}\leq C^{\prime }\max\left\{N^{-\frac{r}{2r+\beta }},h^{-2p}{\left(N+S\right)}^{2p+1}N^{\frac{p+\frac{3}{2}}{2r+\beta }-\left(2p+1\right)}\right\}\log^{4}\frac{24}{\delta }, \end{array}$$

*where $C^{\prime }$ is a constant independent of N,S,δ,h and ⌈N/4⌉ denotes the largest number not exceeding N/4.*

**Corollary** **1.** *Under the same conditions of Theorem 1, if the scaling parameter:*

$$\begin{array}{c} h>{\left(N+S\right)}^{\frac{2p+1}{2p}}N^{\frac{r+p+\frac{3}{2}}{2p(2r+\beta )}}N^{-\frac{2p+1}{2p}}, \end{array}$$

*then for any 0<δ<1, with confidence at least 1-δ:*

(9) $$\begin{array}{c} ∥{\bar{f}}_{T+1,\tilde{D}}-f_{\rho }{∥}_{L^{2}}\leq C^{\prime }N^{-\frac{r}{2r+\beta }}\log^{4}\frac{24}{\delta }. \end{array}$$

**Remark** **2.** *The rate $O\left(N^{-\frac{r}{2r+\beta }}\right)$ in Equation (9) is optimal in the minimax sense for kernel regression problems [13]. When m=1, the result of Equation (9) shows that the kernel gradient descent MEE algorithm (Equation (2)) on a single big data set can achieve the minimax optimal rate for regression. Thus, MEE is a nice alternative of the classical least squares. Meanwhile, the upper bound (Equation (7)) for the number of local machines implies that the performance of the distributed MEE algorithm (Equation (3)) can be as good as the standard MEE algorithm (2) (acting on the whole data set $\tilde{D}$), provided that the subset ${\tilde{D}}_{l}$’s size n+s is not too small.*

**Remark** **3.** If no unlabeled data is engaged in the algorithm (Equation (3)), then S=0 and the upper bound (Equation (7)) for the number of local machines m that ensures the optimal rate is about $O\left(N^{\frac{r-\frac{1}{2}}{2r+\beta }}\right).$ So, when the regularity parameter r in Equation (5) is close to $\frac{1}{2},$ the upper bound $O\left(N^{\frac{r-\frac{1}{2}}{2r+\beta }}\right)$ reduces to a constant and then the distributed algorithm (Equation (3)) will not be feasible in real applications. A similar phenomenon is observed in various distributed algorithms [15,16,17,18]. When the size of unlabeled data S>0, we see from Equation (7) that the upper bound of m keeps growing with the increase of S when the size of labeled data N is fixed. For example, let $\beta >\frac{1}{2}$ and $S=N^{\frac{1}{2r+\beta }}$, then the upper bound in Equation (7) is $O\left(N^{\frac{r}{2r+\beta }}\right)$ and will not be a constant when $r\to \frac{1}{2}.$ Hence, with sufficient unlabeled data $D^{*}$, the distributed algorithm (Equation (3)) will allow more local machines in the distributed method.

**Remark** **4.** *A series of distributed works [15,16,17,18,19] were carried out when the target function $f_{\rho }$ lies in the space $H_{K},$ i.e., the regularization parameter $r>\frac{1}{2}.$ As a byproduct, our work in Theorem 1 does not impose the restriction $r>\frac{1}{2}$ on the distributed algorithm (Equation (3)).*

## 3. Proof of Main Result

In this section we prove our main results in Theorem 1. To this end, we introduce the data-free gradient descent method in $H_{K}$ for the least squares, defined as $f_{1}=0$ and:

$$\begin{array}{c} f_{t+1}=f_{t}-\eta_{t}\int_{X}\int_{X}\left(f_{t}\left(x,x^{\prime }\right)-f_{\rho }\left(x,x^{\prime }\right)\right)K_{\left(x,x^{\prime }\right)}d\rho_{X}\left(x\right)d\rho_{X}\left(x^{\prime }\right),\;t\geq 1. \end{array}$$

Recalling the definition of $L_{K}$, it can be written as:

(10) $$\begin{array}{c} f_{t+1}=f_{t}-\eta_{t}L_{K}\left(f_{t}-f_{\rho }\right)=\left(I-\eta_{t}L_{K}\right)f_{t}+\eta_{t}L_{K}\left(f_{\rho }\right),\;t\geq 1. \end{array}$$

Following the standard decomposition technique in leaning theory, we split the error ${\bar{f}}_{t+1,\tilde{D}}-f_{\rho }$ into the sample error ${\bar{f}}_{t+1,\tilde{D}}-f_{t+1}$ and the approximation error $f_{t+1}-f_{\rho }.$

### 3.1. Approximation Error

Firstly, we estimate the approximation error $∥f_{t+1}-f_{\rho }{∥}_{L^{2}}.$ It has been proven in Reference [20] and shown in the lemmas as follows.

**Lemma** **1.** *Define $\left\{f_{t}\right\}$ by Equation (10) with 0<η≤1. If Equation (5) holds with r>0, there are:*

$$\begin{array}{c} ∥f_{t}-f_{\rho }{∥}_{L^{2}}\leq c_{\phi ,r}t^{-r}, \end{array}$$

*and when $r\geq \frac{1}{2}$:*

$$\begin{array}{c} ∥f_{t}-f_{\rho }{∥}_{K}\leq c_{\phi ,r}t^{-(r-\frac{1}{2})}, \end{array}$$

*where $c_{\phi ,r}=\max\left\{{∥\phi ∥}_{L^{2}}{\left(2r/e\right)}^{r},{∥\phi ∥}_{L^{2}}{\left[\left(2r-1\right)/e\right]}^{r-\frac{1}{2}}\right\}$.*

Moreover, we derive the uniform bound of the sequence $\left\{f_{t}\right\}$ by Equation (10) when $0<r<\frac{1}{2}$, which is useful in our analysis. Here and in the sequel, denote $\pi_{i+1}^{t}\left(L\right)$ as the polynomial operator associated with an operator *L* defined by $\pi_{i+1}^{t}\left(L\right):=\prod_{j=i+1}^{t}\left(I-\eta L\right)$ and $\pi_{t+1}^{t}:=I.$ We use the conventional notation $\sum_{j=T+1}^{T}:=1.$

**Lemma** **2.** *Define $\left\{f_{t}\right\}$ by Equation (10) with 0<η≤1. If Equation (5) holds with $0<r<\frac{1}{2}$, there are:*

(11) $$\begin{array}{c} ∥f_{t}{∥}_{K}\leq d_{\phi ,\eta ,r}t^{\frac{1}{2}-r}, \end{array}$$

*where $d_{\phi ,\eta ,r}$ is defined in the proof.*

**Proof.**  Using Equation (10) iteratively from *t* to 1, then we have that:

$$\begin{array}{c} f_{t+1}=\sum_{i=1}^{t}\eta \pi_{i+1}^{t}\left(L_{K}\right)L_{K}\left(f_{\rho }\right),\;for\;all\;t\geq 1. \end{array}$$

With Equation (5):

(12) $$\begin{array}{cc} ∥f_{t+1}{∥}_{K} & ={∥\sum_{i=1}^{t}\eta \pi_{i+1}^{t}\left(L_{K}\right)L_{K}\left(f_{\rho }\right)∥}_{K}={∥\sum_{i=1}^{t}\eta \pi_{i+1}^{t}\left(L_{K}\right)L_{K}L_{K}^{r}\left(\phi \right)∥}_{K}\\ & =∥\sum_{i=1}^{t}\eta \pi_{i+1}^{t}\left(L_{K}\right)L_{K}^{r+\frac{1}{2}}∥∥L_{K}^{\frac{1}{2}}{\phi ∥}_{K}=∥\sum_{i=1}^{t}\eta \pi_{i+1}^{t}\left(L_{K}\right)L_{K}^{r+\frac{1}{2}}∥{∥\phi ∥}_{L^{2}}. \end{array}$$

Let ${\left\{\sigma_{k}\right\}}_{k=1}^{\infty }$ be the eigenvalues of the operator $L_{K}$ and $0\leq \sigma_{k}\leq 1,k\geq 1$, since $L_{K}$ is positive and $∥L_{K}{∥}_{H_{K}\to H_{K}}\leq 1$, then the norm:

$$\begin{array}{c} ∥\sum_{i=1}^{t}\eta \pi_{i+1}^{t}\left(L_{K}\right)L_{K}^{r+\frac{1}{2}}∥=\underset{k\geq 1}{\sup}\left|\sum_{i=1}^{t}\eta \pi_{i+1}^{t}\left(\sigma_{k}\right)\sigma_{k}^{r+\frac{1}{2}}\right|\leq \eta \underset{a>0}{\sup}\left|\sum_{i=1}^{t-1}\pi_{i+1}^{t}\left(a\right)a^{r+\frac{1}{2}}\right|+∥\eta L_{K}^{r+\frac{1}{2}}∥\\ \leq \eta \underset{a>0}{\sup}\left\{\sum_{i=1}^{t-1}\left[\exp\left\{-\eta a(t-i)\right\}\right]a^{r+\frac{1}{2}}\right\}+\eta \\ \leq \sum_{i=1}^{t-1}\underset{a>0}{\sup}\left\{\eta \left[\exp\left\{-\eta a(t-i)\right\}\right]a^{r+\frac{1}{2}}\right\}+\eta . \end{array}$$

For each i≤t-1, by a simple calculation, we have:

$$\begin{array}{c} \underset{a>0}{\sup}\left\{\left[\exp\left\{-\eta a(t-i)\right\}\right]a^{r+\frac{1}{2}}\right\}=\left\{\left[\exp\left\{-\eta a(t-i)\right\}\right]a^{r+\frac{1}{2}}\right\}{|}_{a=\left(r+\frac{1}{2}\right){\left(\eta (t-i)\right)}^{-1}}\\ =\eta^{\frac{1}{2}-r}{\left(r+\frac{1}{2}\right)}^{r+\frac{1}{2}}\exp\left\{-(r+\frac{1}{2})\right\}{\left(t-i\right)}^{-(r+\frac{1}{2})}\leq {\left(t-i\right)}^{-(r+\frac{1}{2})}. \end{array}$$

Thus, we have:

$$\begin{array}{c} ∥\sum_{i=1}^{t}\eta \pi_{i+1}^{t}\left(L_{K}\right)L_{K}^{r+\frac{1}{2}}∥\leq \eta \sum_{i=1}^{t-1}{\left(t-i\right)}^{-(r+\frac{1}{2})}+\eta =\eta \sum_{i=1}^{t-1}i^{-(r+\frac{1}{2})}+\eta . \end{array}$$

By the elementary inequality $\sum_{i=1}^{t}t^{-\theta }\leq \frac{t^{1-\theta }}{1-\theta }$ with 0<θ<1, it follows that:

$$\begin{array}{c} ∥\sum_{i=1}^{t}\eta \pi_{i+1}^{t}\left(L_{K}\right)L_{K}^{r+\frac{1}{2}}∥\leq \eta \left(\frac{1}{1/2-r}+1\right)t^{\frac{1}{2}-r}=\eta \left(\frac{3/2-r}{1/2-r}\right)t^{\frac{1}{2}-r}. \end{array}$$

Together with Equation (12), then the proof is completed by taking $d_{\phi ,\eta ,r}:=\eta \left(\frac{3/2-r}{1/2-r}\right){∥\phi ∥}_{L^{2}}.$ □

### 3.2. Sample Error

Define the empirical operator $L_{K,D}:H_{K}\to H_{K}$ by:

$$\begin{array}{c} L_{K,D}:=\frac{1}{N^{2}}\sum_{\begin{array}{c} (x_{i},y_{i})\in D\\ (x_{j},y_{j})\in D \end{array}}{\langle \cdot ,K_{\left(x_{i},x_{j}\right)}\rangle }_{K}K_{\left(x_{i},x_{j}\right)}, \end{array}$$

and for any $f\in H_{K}$:

$$\begin{array}{c} L_{K,D}\left(f\right)=\frac{1}{N^{2}}\sum_{\begin{array}{c} (x_{i},y_{i})\in D\\ (x_{j},y_{j})\in D \end{array}}{\langle f,K_{\left(x_{i},x_{j}\right)}\rangle }_{K}K_{\left(x_{i},x_{j}\right)}=\frac{1}{N^{2}}\sum_{\begin{array}{c} (x_{i},y_{i})\in D\\ (x_{j},y_{j})\in D \end{array}}f\left(x_{i},x_{j}\right)K_{\left(x_{i},x_{j}\right)}. \end{array}$$

Then, the MEE gradient descent algorithm (Equation (2)) on $\tilde{D}$ can be written as:

(13) $$\begin{array}{c} f_{t+1,\tilde{D}}=\left[I-\eta L_{K,\tilde{D}}\right]\left(f_{t,\tilde{D}}\right)+\eta f_{\rho ,\tilde{D}}+\eta E_{t,\tilde{D}}, \end{array}$$

where:

(14) $$E_{t,\tilde{D}}=\frac{1}{{\left(N+S\right)}^{2}}\sum_{\begin{array}{c} \left(x_{i},y_{i}\right)\in \tilde{D}\\ \left(x_{j},y_{j}\right)\in \tilde{D} \end{array}}\left(G^{\prime }\left(\frac{{\left[y_{i}-y_{j}-f_{t,\tilde{D}}\left(x_{i},x_{j}\right)\right]}^{2}}{h^{2}}\right)-G^{\prime }\left(0\right)\right)\left(f_{t,\tilde{D}}\left(x_{i},x_{j}\right)-y_{i}+y_{j}\right)K_{\left(x_{i},x_{j}\right)},$$

and:

$$\begin{array}{c} f_{\rho ,\tilde{D}}=\frac{1}{{\left(N+S\right)}^{2}}\sum_{\begin{array}{c} \left(x_{i},y_{i}\right)\in \tilde{D}\\ \left(x_{j},y_{j}\right)\in \tilde{D} \end{array}}\left(y_{i}-y_{j}\right)K_{\left(x_{i},x_{j}\right)}. \end{array}$$

In the sequel, denote:

$$\begin{array}{c} B_{\tilde{D},\lambda }=∥{\left(L_{K,\tilde{D}}+\lambda I\right)}^{-1}\left(L_{K}+\lambda \right)∥,\\ C_{\tilde{D},\lambda }=∥{\left(L_{K}+\lambda I\right)}^{-\frac{1}{2}}\left(L_{K}-L_{K,\tilde{D}}\right)∥,\\ D_{\tilde{D},\lambda }=∥\frac{1}{m}\sum_{l=1}^{m}{\left(L_{K}+\lambda I\right)}^{-\frac{1}{2}}\left(L_{K}-L_{K,{\tilde{D}}_{l}}\right)∥,\\ F_{\tilde{D},\lambda }={∥\frac{1}{m}\sum_{l=1}^{m}{\left(L_{K}+\lambda I\right)}^{-\frac{1}{2}}\left[f_{\rho ,{\tilde{D}}_{l}}-L_{K}\left(f_{\rho }\right)\right]∥}_{K},\\ G_{\tilde{D},\lambda }={∥{\left(L_{K}+\lambda I\right)}^{-\frac{1}{2}}\left(L_{K}f_{\rho }-f_{\rho ,\tilde{D}}\right)∥}_{K}. \end{array}$$

With these preliminaries in place, we now turn to the estimates of the sample error ${\bar{f}}_{t+1,\tilde{D}}-f_{t+1}$ presented in the following Lemma, whose proof can be found in the Appendix. Here and in the sequel, we use the conventional notation $\sum_{i=1}^{t}{\left(t-i\right)}^{-1}:=\sum_{i=1}^{t-1}{\left(t-i\right)}^{-1}+1.$

**Lemma** **3.** Let λ>0 and $0<\eta <\min\{C_{G}^{-1},1\},$ for any $f^{*}\in H_{K},$ there holds:

(15) $$\begin{array}{c} ∥{\bar{f}}_{T+1,\tilde{D}}-f_{T+1}{∥}_{L^{2}}\leq term\;1+term\;2+c_{p,M}{\left|N+S\right|}^{2p+1}N^{-(2p+1)}T^{p+3/2}h^{-2p}, \end{array}$$

*where the constant $c_{p,M}=2^{4p+2}c_{p}C_{G}^{2p+1}M^{2p+1}$:*

$$\begin{array}{cc} term\;1 & =\underset{1\leq l\leq m}{\sup}\sum_{i=1}^{T}\left({\left(T-i\right)}^{-1}+\eta \lambda \right)C_{{\tilde{D}}_{l},\lambda }\times \{\sum_{s=1}^{i-1}\left({\left(i-s-1\right)}^{-1}+\lambda \eta \right){∥f_{s}-f^{*}∥}_{K}B_{{\tilde{D}}_{l},\lambda }C_{{\tilde{D}}_{l},\lambda }\lambda^{-\frac{1}{2}}\\ & +\left(1+\lambda \eta i\right)B_{{\tilde{D}}_{l},\lambda }(C_{{\tilde{D}}_{l},\lambda }∥f^{*}{∥}_{K}+G_{{\tilde{D}}_{l},\lambda })\lambda^{-\frac{1}{2}}+c_{p,M}{\left|N+S\right|}^{2p+1}N^{-(2p+1)}i^{p+1/2}h^{-2p}\}, \end{array}$$

*and:*

$$\begin{array}{c} term\;2=\sum_{i=1}^{T}\left({\left(T-i\right)}^{-1}+\eta \lambda \right)D_{\tilde{D},\lambda }∥f_{i}-f^{*}{∥}_{K}+\left(1+\lambda \eta T\right)(D_{\tilde{D},\lambda }∥f^{*}{∥}_{K}+F_{\tilde{D},\lambda }). \end{array}$$

With the help of Lemma above, to bound the sample error $∥{\bar{f}}_{T+1,\tilde{D}}-f_{T+1}{∥}_{L^{2}},$ we first need to estimate the quantities the quantities $B_{\tilde{D},\lambda },$ $C_{\tilde{D},\lambda },$ $D_{\tilde{D},\lambda }$ $F_{\tilde{D}\lambda }$ and $G_{\tilde{D},\lambda }.$ Denote $A_{D,\lambda }:=\frac{1}{\left\lceil |D|/4\right\rceil \sqrt{\lambda }}+\sqrt{\frac{N(\lambda )}{\left\lceil |D|/4\right\rceil }}$ (|D| is the cardinality of *D*). In previous work [19,21,22,23], we have foundnd that each of the following inequality holds with confidence at least 1-δ:

(16) $$\begin{array}{c} B_{\tilde{D},\lambda }\leq 2{\left(\frac{2A_{\tilde{D},\lambda }\log\frac{2}{\delta }}{\sqrt{\lambda }}\right)}^{2}+2,\;C_{\tilde{D},\lambda }\leq 2A_{\tilde{D},\lambda }\log\frac{2}{\delta },\;D_{{\tilde{D}}_{,}\lambda }\leq 2A_{\tilde{D},\lambda }\log\frac{2}{\delta }\\ F_{\tilde{D},\lambda }\leq 16MA_{D,\lambda }\log\frac{4}{\delta },\;and\;G_{\tilde{D},\lambda }\leq 16MA_{D,\lambda }\log\frac{4}{\delta }. \end{array}$$

By Lemma 3, we also see that the function $f^{*}$ is crucial to determine $∥{\bar{f}}_{T+1,\tilde{D}}-f_{T+1}∥.$ To get a tight bound for the learning error, we should choose an appropriate $f^{*}\in H_{\tilde{K}}$ according to the regularity of the target function. When $r\geq \frac{1}{2},$ $f_{\rho }\in H_{K}$ and we take $f^{*}=f_{\rho }.$ When $0<r<\frac{1}{2},$ $f_{\rho }$ is out of the space $H_{K}$ and we let $f^{*}=0.$

Now, we give the first main result when the target function $f_{\rho }$ is out of $H_{K}$ with $0<r<\frac{1}{2}.$

**Theorem** **2.** *Assume Equation (5) for $0<r<\frac{1}{2}.$ Let $0<\eta <\min\{1,C_{G}^{-1}\},$T∈N and $\lambda =T^{-1}.$ Then, for any 0<δ<1, with probability at least 1-δ, there holds:*

(17) $$\begin{array}{cc} ∥{\bar{f}}_{T+1,\tilde{D}}-f_{\rho }{∥}_{L^{2}} & \leq C^{*}\{T^{-r}+\log^{2}\left(T\right)J_{D,\tilde{D},\lambda }\log^{4}\frac{24m}{\delta }+\left(\log\left(T\right)A_{\tilde{D},\lambda }\lambda^{r-\frac{1}{2}}+A_{D,\lambda }\right)\log\frac{16}{\delta }\\ & {+|N+S|}^{2p+1}N^{-(2p+1)}h^{-2p}\left(T^{p+3/2}+T^{p+\frac{1}{2}}\log\left(T\right)\underset{1\leq l\leq k}{\sup}A_{{\tilde{D}}_{l},\lambda }\right)\log\frac{2}{\delta }\}, \end{array}$$

where $C^{*}$ is a constant given in the proof, $J_{D,\tilde{D},\lambda }=\underset{1\leq l\leq m}{\sup}\left({\left(\frac{A_{{\tilde{D}}_{l},\lambda }}{\sqrt{\lambda }}\right)}^{2}+1\right)\left(A_{{\tilde{D}}_{l},\lambda }^{2}\lambda^{r-1}+A_{{\tilde{D}}_{l},\lambda }A_{D_{l},\lambda }\lambda^{-\frac{1}{2}}\right).$

**Proof.**  Decompose $∥{\bar{f}}_{T+1,\tilde{D}}-f_{\rho }{∥}_{L^{2}}$ into:

$$\begin{array}{c} ∥{\bar{f}}_{T+1,\tilde{D}}-f_{\rho }{∥}_{L^{2}}\leq ∥{\bar{f}}_{T+1,\tilde{D}}-f_{T+1}{∥}_{L^{2}}+{∥f_{T+1}-f_{\rho }∥}_{L^{2}}. \end{array}$$

The estimate of $∥f_{T+1}-f_{\rho }{∥}_{L^{2}}$ is presented in Lemma 1. We only need to handle $∥{\bar{f}}_{T+1,\tilde{D}}-f_{T+1}{∥}_{L^{2}}$ by Lemma 3.

For any 0<s≤T-1 and $\lambda =T^{-1}$, by Equation (11), we have $∥f_{s}{∥}_{K}\leq d_{\phi ,\eta ,r}s^{\frac{1}{2}-r}\leq d_{\phi ,\eta ,r}\lambda^{r-\frac{1}{2}}$. Take $f^{*}=0$ in Lemma 3, then:

$$\begin{array}{cc} term\;1 & \leq \left(1+d_{\phi ,\eta ,r}+c_{p,M}\right)\underset{1\leq l\leq m}{\sup}\sum_{i=1}^{T}\left({\left(T-i\right)}^{-1}+\eta \lambda \right)C_{{\tilde{D}}_{l},\lambda }\times \{\sum_{s=1}^{i-1}\left({\left(i-s-1\right)}^{-1}+\lambda \eta \right)B_{{\tilde{D}}_{l},\lambda }C_{{\tilde{D}}_{l},\lambda }\lambda^{r-1}\\ & +\left(1+\lambda \eta i\right)B_{{\tilde{D}}_{l},\lambda }\left(C_{{\tilde{D}}_{l},\lambda }\lambda^{r-\frac{1}{2}}+G_{{\tilde{D}}_{l},\lambda }\right)\lambda^{-\frac{1}{2}}+{\left|N+S\right|}^{2p+1}N^{-(2p+1)}i^{p+1/2}h^{-2p}\}, \end{array}$$

and:

$$\begin{array}{cc} term\;2 & \leq \left(1+d_{\phi ,\eta ,r}\right)\left\{\sum_{i=1}^{T}\left({\left(T-i\right)}^{-1}+\eta \lambda \right)D_{\tilde{D},\lambda }\lambda^{r-\frac{1}{2}}+\left(1+\lambda \eta T\right)\left(D_{\tilde{D},\lambda }\lambda^{r-\frac{1}{2}}+F_{\tilde{D},\lambda }\right)\right\}. \end{array}$$

Noticing the elementary inequality $\sum_{s=1}^{i}i^{-1}\leq 2\log\left(i\right),$ then:

$$\begin{array}{c} \sum_{s=1}^{i-1}\left({\left(i-s-1\right)}^{-1}+\lambda \eta \right)\leq \sum_{s=1}^{i-1}\left({\left(i-s-1\right)}^{-1}+T^{-1}\right)\leq 4\log\left(i\right), \end{array}$$

$$\begin{array}{c} \sum_{i=1}^{T}\left({\left(T-i\right)}^{-1}+\eta \lambda \right)\sum_{s=1}^{i-1}\left({\left(i-s-1\right)}^{-1}+\lambda \eta \right)\leq 4\sum_{i=1}^{T}\left({\left(T-i\right)}^{-1}+T^{-1}\right)\log\left(i\right)\\ \leq 16\sum_{i=1}^{T}\frac{\log(i)}{T-i}\leq 16\log\left(T\right)\sum_{i=1}^{T}\frac{1}{T-i}=16\log\left(T\right)\sum_{i=1}^{T-1}i^{-1}\leq 32\log^{2}\left(T\right), \end{array}$$

$$\begin{array}{c} \sum_{i=1}^{T}\left({\left(T-i\right)}^{-1}+\eta \lambda \right)\left(1+\lambda \eta i\right)\leq \sum_{i=1}^{T}\left({\left(T-i\right)}^{-1}+\eta T^{-1}\right)\left(1+T^{-1}\eta i\right)\\ \leq 2\sum_{i=1}^{T}\left({\left(T-i\right)}^{-1}+1\right)\leq 8\log\left(T\right), \end{array}$$

and:

$$\begin{array}{c} \sum_{i=1}^{T}\left({\left(T-i\right)}^{-1}+\eta \lambda \right)\leq 4\log\left(T\right), \end{array}$$

$$\begin{array}{c} \sum_{i=1}^{T}\left({\left(T-i\right)}^{-1}+\eta \lambda \right)i^{p+1/2}\leq \sum_{i=1}^{T}\left({\left(T-i\right)}^{-1}+\eta \lambda \right)T^{p+1/2}\leq 4T^{p+1/2}\log\left(T\right). \end{array}$$

Plugging the above inequalities into *term* 1 and *term* 2, then:

(18) $$\begin{array}{cc} term\;1\leq & \underset{1\leq l\leq m}{\sup}C_{1}(\log^{2}\left(T\right)B_{{\tilde{D}}_{l},\lambda }C_{{\tilde{D}}_{l},\lambda }^{2}\lambda^{r-1}+\log\left(T\right)B_{{\tilde{D}}_{l},\lambda }C_{{\tilde{D}}_{l},\lambda }^{2}\lambda^{r-1}\\ & +\log\left(T\right)B_{{\tilde{D}}_{l},\lambda }C_{{\tilde{D}}_{l},\lambda }G_{{\tilde{D}}_{l},\lambda }\lambda^{-\frac{1}{2}}+{\left|N+S\right|}^{2p+1}N^{-(2p+1)}T^{p+1/2}\log\left(T\right)C_{{\tilde{D}}_{l},\lambda }h^{-2p}), \end{array}$$

and:

(19) $$\begin{array}{c} term\;2\leq C_{2}\left(\log\left(T\right)D_{\tilde{D},\lambda }\lambda^{r-\frac{1}{2}}+F_{\tilde{D},\lambda }\right), \end{array}$$

where $C_{1}=32\left(1+d_{\phi ,\eta ,r}+c_{p,M}\right)$ and $C_{2}=6\left(1+d_{\phi ,\eta ,r}\right)$.

By Equation (16), for any fixed l, there exist three subsets with measure at least 1-δ such that:

$$\begin{array}{c} B_{{\tilde{D}}_{l},\lambda }\leq 2{\left(\frac{2A_{{\tilde{D}}_{l},\lambda }\log\frac{2}{\delta }}{\sqrt{\lambda }}\right)}^{2}+2,\;C_{{\tilde{D}}_{l},\lambda }\leq 2A_{{\tilde{D}}_{l},\lambda }\log\frac{2}{\delta }, \end{array}$$

and:

$$\begin{array}{c} G_{{\tilde{D}}_{l},\lambda }\leq 16MA_{D_{l},\lambda }\log\frac{4}{\delta }. \end{array}$$

Thus, for any fixed l, with confidence at least 1-3δ, there holds:

$$\begin{array}{c} B_{{\tilde{D}}_{l},\lambda }C_{{\tilde{D}}_{l},\lambda }^{2}\lambda^{r-1}\leq 32\left({\left(\frac{A_{{\tilde{D}}_{l},\lambda }}{\sqrt{\lambda }}\right)}^{2}+1\right)A_{{\tilde{D}}_{l},\lambda }^{2}\lambda^{r-1}\log^{4}\frac{2}{\delta }, \end{array}$$

and:

$$\begin{array}{c} B_{{\tilde{D}}_{l},\lambda }C_{{\tilde{D}}_{l},\lambda }G_{{\tilde{D}}_{l},\lambda }\lambda^{-\frac{1}{2}}\leq 256M\left({\left(\frac{A_{{\tilde{D}}_{l},\lambda }}{\sqrt{\lambda }}\right)}^{2}+1\right)A_{{\tilde{D}}_{l},\lambda }A_{D_{l},\lambda }\lambda^{-\frac{1}{2}}\log^{3}\frac{2}{\delta }\log\frac{4}{\delta }. \end{array}$$

Therefore, with confidence at least 1-3mδ, there holds:

$$\begin{array}{c} \underset{1\leq l\leq m}{\sup}B_{{\tilde{D}}_{l},\lambda }C_{{\tilde{D}}_{l},\lambda }^{2}\lambda^{r-1}\leq 32\left({\left(\frac{A_{{\tilde{D}}_{l},\lambda }}{\sqrt{\lambda }}\right)}^{2}+1\right)A_{{\tilde{D}}_{l},\lambda }^{2}\lambda^{r-1}\log^{4}\frac{2}{\delta }, \end{array}$$

and:

$$\begin{array}{c} \underset{1\leq l\leq m}{\sup}B_{{\tilde{D}}_{l},\lambda }C_{{\tilde{D}}_{l},\lambda }G_{{\tilde{D}}_{l},\lambda }\lambda^{-\frac{1}{2}}\leq 256M\left({\left(\frac{A_{{\tilde{D}}_{l},\lambda }}{\sqrt{\lambda }}\right)}^{2}+1\right)A_{{\tilde{D}}_{l},\lambda }A_{D_{l},\lambda }\lambda^{-\frac{1}{2}}\log^{3}\frac{2}{\delta }\log\frac{4}{\delta }. \end{array}$$

Thus, by Equation (18), it follows that with confidence at least 1-δ/2 by scaling 3mδ to δ/2, there holds:

$$\begin{array}{cc} term\;1 & \leq C_{3}\underset{1\leq l\leq m}{\sup}(\log^{2}\left(T\right)\left({\left(\frac{A_{{\tilde{D}}_{l},\lambda }}{\sqrt{\lambda }}\right)}^{2}+1\right)\left(A_{{\tilde{D}}_{l},\lambda }^{2}\lambda^{r-1}+A_{{\tilde{D}}_{l},\lambda }A_{D_{l},\lambda }\lambda^{-\frac{1}{2}}\right)\log^{4}\frac{24m}{\delta }\\ & {+|N+S|}^{2p+1}N^{-(2p+1)}T^{p+1/2}\log\left(T\right)A_{{\tilde{D}}_{l},\lambda }h^{-2p}), \end{array}$$

where $C_{3}=C_{1}\left(256M+64\right).$

Similarly, with confidence at least 1-2δ such that:

$$\begin{array}{c} D_{\tilde{D},\lambda }\leq 2A_{\tilde{D},\lambda }\log\frac{2}{\delta },\;and\;F_{\tilde{D},\lambda }\leq 16MA_{D,\lambda }\log\frac{4}{\delta }. \end{array}$$

By Equation (19), it follows that with confidence at least 1-δ/2 by scaling 2δ to δ/2:

$$\begin{array}{c} term\;2\leq C_{4}\left(\log\left(T\right)A_{\tilde{D},\lambda }\lambda^{r-\frac{1}{2}}+A_{D,\lambda }\right)\log\frac{16}{\delta }, \end{array}$$

where $C_{4}=C_{2}\left(16M+2\right).$ Together with Lemma 1, we obtain the desired bound (Equation (17)) with $C^{*}=c_{\phi ,r}+C_{3}+C_{4}+c_{p,M}.$ □

Next, we give the result when the target function $f_{\rho }$ is in $H_{K}$ with $r\geq \frac{1}{2}.$

**Theorem** **3.** *Assume Equation (1) for $r\geq \frac{1}{2}.$ Let $0<\eta <\min\{1,C_{G}^{-1}\},$T∈N and $\lambda =T^{-1}.$ Then, for any 0<δ<1, with probability at least 1-δ, there holds:*

(20) $$\begin{array}{cc} ∥{\bar{f}}_{T+1,\tilde{D}}-f_{\rho }{∥}_{L^{2}} & \leq C^{*}\{T^{-r}+\log^{2}\left(T\right)K_{D,\tilde{D},\lambda }\log^{4}\frac{24m}{\delta }+\left(\log\left(T\right)A_{\tilde{D},\lambda }+A_{D,\lambda }\right)\log\frac{16}{\delta }\\ & {+|N+S|}^{2p+1}N^{-(2p+1)}h^{-2p}\left(T^{p+3/2}+T^{p+\frac{1}{2}}\log\left(T\right)\underset{1\leq l\leq k}{\sup}A_{{\tilde{D}}_{l},\lambda }\right)\log\frac{2}{\delta }\}, \end{array}$$

where $K_{D,\tilde{D},\lambda }=\sup_{1\leq l\leq m}\left({\left(\frac{A_{{\tilde{D}}_{l},\lambda }}{\sqrt{\lambda }}\right)}^{2}+1\right)\left(A_{{\tilde{D}}_{l},\lambda }^{2}+A_{{\tilde{D}}_{l},\lambda }A_{D_{l},\lambda }\right)\lambda^{-\frac{1}{2}}$ and $C^{*}$ is a constant given in the proof.

The proof is similar to that of Theorem 2. Here we omit it.

With these preliminaries in place, we can prove our main result in Theorem 1.

**Proof** **of** **Theorem** **1.** We first prove Equation (8) by Theorem 2 when $0<r<\frac{1}{2}.$ Let $T={\left\lceil |D|/4\right\rceil }^{\frac{1}{2r+\beta }}$ and $\lambda =T^{-1}$. Notice that |D|=N, $|\tilde{D}|=N+S$ and $m|D_{l}\left|=|D|,m\right|{\tilde{D}}_{l}\left|=\right|\tilde{D}|$ for 1≤l≤m, with $r+\beta >\frac{1}{2}$ and Equation (7), we obtain that:

$$\begin{array}{cc} A_{D,\lambda } & ={\left\lceil |D|/4\right\rceil }^{-1+\frac{1}{4r+2\beta }}+\sqrt{C_{0}}{\left\lceil |D|/4\right\rceil }^{-\frac{1}{2}+\frac{\beta }{4r+2\beta }}\\ & \leq \left(\sqrt{C_{0}}+1\right){\left\lceil |D|/4\right\rceil }^{-\frac{r}{2r+\beta }}\leq \sqrt{5}\left(\sqrt{C_{0}}+1\right){\left|D\right|}^{-\frac{r}{2r+\beta }}\\ & =\sqrt{5}\left(\sqrt{C_{0}}+1\right)N^{-\frac{r}{2r+\beta }}, \end{array}$$

$$\begin{array}{cc} A_{\tilde{D},\lambda } & =\left\lceil \right|\tilde{D}{\left|/4\right\rceil }^{-1}{\left[|D|/4\right]}^{\frac{1}{4r+2\beta }}+\sqrt{C_{0}}\left\lceil \right|\tilde{D}{\left|/4\right\rceil }^{-\frac{1}{2}}{\left\lceil |D|/4\right\rceil }^{\frac{\beta }{4r+2\beta }}\\ & \leq \sqrt{5}\left(\sqrt{C_{0}}+1\right)\left(\right|\tilde{D}{|}^{-1}{\left|D\right|}^{\frac{1}{4r+2\beta }}+|\tilde{D}{|}^{-\frac{1}{2}}{\left|D\right|}^{\frac{\beta }{4r+2\beta }})\\ & =\sqrt{5}\left(\sqrt{C_{0}}+1\right){\left(|N+S\right|}^{-1}N^{\frac{1}{4r+2\beta }}{+|N+S|}^{-\frac{1}{2}}N^{\frac{\beta }{4r+2\beta }}), \end{array}$$

$$\begin{array}{cc} A_{D_{l},\lambda } & =\left\lceil \right|D_{l}{\left|/4\right\rceil }^{-1}{\left\lceil |D|/4\right\rceil }^{\frac{1}{4r+2\beta }}+\sqrt{C_{0}}\left\lceil \right|D_{l}{\left|/4\right\rceil }^{-\frac{1}{2}}{\left\lceil |D|/4\right\rceil }^{\frac{\beta }{4r+2\beta }}\\ & \leq \sqrt{5}\left(\sqrt{C_{0}}+1\right){\left(m|D\right|}^{-1+\frac{1}{4r+2\beta }}+m^{\frac{1}{2}}{\left|D\right|}^{-\frac{1}{2}+\frac{\beta }{4r+2\beta }})\\ & =\sqrt{5}\left(\sqrt{C_{0}}+1\right)\left(mN^{-1+\frac{1}{4r+2\beta }}+m^{\frac{1}{2}}N^{-\frac{1}{2}+\frac{\beta }{4r+2\beta }}\right), \end{array}$$

and:

$$\begin{array}{cc} A_{{\tilde{D}}_{l},\lambda } & \leq \sqrt{5}\left(\sqrt{C_{0}}+1\right)\left(\right|{\tilde{D}}_{l}{|}^{-1}{\left|D\right|}^{\frac{1}{4r+2\beta }}+|{\tilde{D}}_{l}{|}^{-\frac{1}{2}}{\left|D\right|}^{\frac{\beta }{4r+2\beta }})\\ & =\sqrt{5}\left(\sqrt{C_{0}}+1\right)\left(m\right|\tilde{D}{|}^{-1}{\left|D\right|}^{\frac{1}{4r+2\beta }}+m^{\frac{1}{2}}|\tilde{D}{|}^{-\frac{1}{2}}{\left|D\right|}^{\frac{\beta }{4r+2\beta }})\\ & \leq 2\sqrt{5}\left(\sqrt{C_{0}}+1\right)m^{\frac{1}{2}}|\tilde{D}{|}^{-\frac{1}{2}}{\left|D\right|}^{\frac{\beta }{4r+2\beta }}. \end{array}$$

Thus:

$$\begin{array}{cc} \frac{A_{{\tilde{D}}_{l},\lambda }}{\sqrt{\lambda }} & \leq \sqrt{5}\left(\sqrt{C_{0}}+1\right)\left(m\right|\tilde{D}{|}^{-1}{\left|D\right|}^{\frac{1}{4r+2\beta }}+m^{\frac{1}{2}}|\tilde{D}{|}^{-\frac{1}{2}}{\left|D\right|}^{\frac{\beta }{4r+2\beta }}{)\lceil |D|/4\rceil }^{\frac{1}{2(2r+\beta )}}\\ & \leq 2\sqrt{5}\left(\sqrt{C_{0}}+1\right)m^{\frac{1}{2}}|\tilde{D}{|}^{-\frac{1}{2}}{\left|D\right|}^{\frac{\beta +1}{4r+2\beta }}\\ & =2\sqrt{5}\left(\sqrt{C_{0}}+1\right)m^{\frac{1}{2}}{\left|N+S\right|}^{-\frac{1}{2}}N^{\frac{\beta +1}{4r+2\beta }}\leq 2\sqrt{5}\left(\sqrt{C_{0}}+1\right). \end{array}$$

It follows for l=1,⋯,m:

$$\begin{array}{c} {\left(\frac{A_{{\tilde{D}}_{l},\lambda }}{\sqrt{\lambda }}\right)}^{2}+1\leq 20{\left(\sqrt{C_{0}}+1\right)}^{2}+1, \end{array}$$

$$\begin{array}{cc} A_{{\tilde{D}}_{l},\lambda }^{2}\lambda^{r-1} & \leq 10{\left(\sqrt{C_{0}}+1\right)}^{2}(m^{2}|\tilde{D}{|}^{-2}{\left|D\right|}^{\frac{1}{2r+\beta }}+m|\tilde{D}{|}^{-1}{\left|D\right|}^{\frac{s}{2r+\beta }}{)|D|}^{\frac{1-r}{2r+\beta }}\\ & =10{\left(\sqrt{C_{0}}+1\right)}^{2}(m^{2}|\tilde{D}{|}^{-2}{\left|D\right|}^{\frac{2}{2r+\beta }}+m|\tilde{D}{|}^{-1}{\left|D\right|}^{\frac{1+\beta }{2r+\beta }}{)|D|}^{-\frac{r}{2r+\beta }}\\ & \leq 20{\left(\sqrt{C_{0}}+1\right)}^{2}{\left|D\right|}^{-\frac{r}{2r+s}}/\log^{6}\left|D\right|\\ & =20{\left(\sqrt{C_{0}}+1\right)}^{2}N^{-\frac{r}{2r+s}}/\log^{6}N, \end{array}$$

$$\begin{array}{cc} A_{{\tilde{D}}_{l},\lambda }A_{D_{l},\lambda }\lambda^{-\frac{1}{2}} & \leq 10{\left(\sqrt{C_{0}}+1\right)}^{2}m^{\frac{1}{2}}|\tilde{D}{|}^{-\frac{1}{2}}{\left|D\right|}^{\frac{\beta }{4r+2\beta }}{\left(m|D\right|}^{-1+\frac{1}{4r+2\beta }}+m^{\frac{1}{2}}{\left|D\right|}^{-\frac{1}{2}+\frac{\beta }{4r+2\beta }}{)|D|}^{\frac{1}{4r+2\beta }}\\ & \leq 10{\left(\sqrt{C_{0}}+1\right)}^{2}(m^{\frac{3}{2}}|\tilde{D}{|}^{-\frac{1}{2}}{\left|D\right|}^{-\frac{\beta +4r-2}{4r+2\beta }}+m|\tilde{D}{|}^{-\frac{1}{2}}{\left|D\right|}^{\frac{\beta +1-2r}{4r+2\beta }})\\ & \leq 20{\left(\sqrt{C_{0}}+1\right)}^{2}{\left|D\right|}^{-\frac{r}{2r+\beta }}/\log^{6}\left|D\right|\\ & =20{\left(\sqrt{C_{0}}+1\right)}^{2}N^{-\frac{r}{2r+\beta }}/\log^{6}N. \end{array}$$

Thus, by the above estimates:

$$\begin{array}{c} J_{D,\tilde{D},\lambda }\leq 40\left(20{\left(\sqrt{C_{0}}+1\right)}^{2}+1\right){\left(\sqrt{C_{0}}+1\right)}^{2}N^{-\frac{r}{2r+\beta }}/\log^{6}N \end{array}$$

Thus:

$$\begin{array}{c} \log^{2}\left(T\right)J_{D,\tilde{D},\lambda }\log^{4}\frac{24m}{\delta }\leq 2^{4}\log^{2}\left(T\right)J_{D,\tilde{D},\lambda }\left(\log^{4}m\right)\log^{4}\frac{24}{\delta }\\ \leq 2^{4}{\left(2r+\beta \right)}^{-2}\left(\log^{2}N\right)J_{D,\tilde{D},\lambda }\left(\log^{4}m\right)\log^{4}\frac{24}{\delta }\\ \leq 2^{4}{\left(2r+\beta \right)}^{-2}\left(\log^{6}N\right)J_{D,\tilde{D},\lambda }\log^{4}\frac{24}{\delta }\\ \leq 2^{10}{\left(2r+\beta \right)}^{-2}\left(20{\left(\sqrt{C_{0}}+1\right)}^{2}+1\right){\left(\sqrt{C_{0}}+1\right)}^{2}{\left|D\right|}^{-\frac{r}{2r+\beta }}\log^{4}\frac{24}{\delta }\\ =2^{10}{\left(2r+\beta \right)}^{-2}\left(20{\left(\sqrt{C_{0}}+1\right)}^{2}+1\right){\left(\sqrt{C_{0}}+1\right)}^{2}N^{-\frac{r}{2r+\beta }}\log^{4}\frac{24}{\delta }, \end{array}$$

and:

$$\begin{array}{cc} \log\left(T\right)A_{\tilde{D},\lambda }\lambda^{r-\frac{1}{2}} & \leq {\left(2r+\beta \right)}^{-1}\sqrt{5}\left(\sqrt{C_{0}}+1\right)\log|D|(|\tilde{D}{|}^{-1}{\left|D\right|}^{\frac{1}{2r+\beta }}+|\tilde{D}{|}^{-\frac{1}{2}}{\left|D\right|}^{\frac{\beta +1}{4r+2\beta }}{)|D|}^{-\frac{r}{2r+\beta }}\\ & \leq 2\sqrt{5}{\left(2r+\beta \right)}^{-1}\left(\sqrt{C_{0}}+1\right){\left|D\right|}^{-\frac{r}{2r+\beta }}=2\sqrt{5}{\left(2r+\beta \right)}^{-1}\left(\sqrt{C_{0}}+1\right)N^{-\frac{r}{2r+\beta }}. \end{array}$$

Putting the above estimates into Theorem 2, we have the desired conclusion (Equation (8)) with:

$$C^{\prime }=C^{*}\left(2^{10}{\left(2r+\beta \right)}^{-2}\left(20{\left(\sqrt{C_{0}}+1\right)}^{2}+1\right){\left(\sqrt{C_{0}}+1\right)}^{2}+2\sqrt{5}{\left(2r+\beta \right)}^{-1}\left(\sqrt{C_{0}}+1\right)+\sqrt{5}\left(\sqrt{C_{0}}+2\right)\right).$$

When $r\geq \frac{1}{2},$ we apply Theorem 3 and take the same proof procedure a above. Then, the conclusion (Equation (8)) can be obtained. The proof is completed. □

## 4. Simulation and Conclusions

In this section, we provide the simulation to verify our theoretical statements. We assume that the inputs $\left\{x_{i}\right\}$ are independently drawn according to the uniform distribution on [0,1]. Consider the regression model $y_{i}=g_{\rho }\left(x_{i}\right)+\varepsilon_{i},i=1,\cdots ,N$, where $\varepsilon_{i}$ is the independent Gaussian noise $N(0,0.1^{2})$ and:

$$g_{\rho }\left(x\right)=\left\{\begin{array}{cc} x, & \mathrm{if}\;0<x\leq 0.5,\\ 1-x, & \mathrm{if}\;0.5<x\leq 1. \end{array}\right.\;$$

Define the pairwise kernel $K:X^{2}\times X^{2}\to R$ by $K\left(\left(x,u\right),\left(x^{\prime },u^{\prime }\right)\right):=K_{1}\left(x,x^{\prime }\right)+K_{1}\left(u,u^{\prime }\right)-K_{1}\left(x,u^{\prime }\right)-K_{1}\left(x^{\prime },u\right)$ where:

$$\begin{array}{c} K_{1}\left(x,x^{\prime }\right)=1+\min\left\{x,x^{\prime }\right\}. \end{array}$$

**Figure 1:**
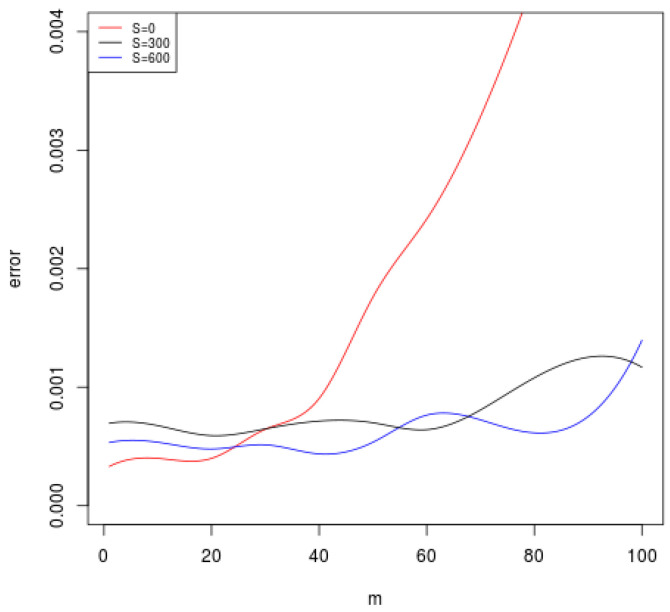
The mean square errors for the size of unlabeled data S∈{0,300,600} as the number of local machines *m* varies.

We apply the kernel *K* to the distributed algorithm (Equation (3)). In Figure 1, we plot the mean squared error of Equation (3) for N=600 and S=0,300,600 when the number of local machines *m* varies. Note that S=0, and it is a standard distributed MEE algorithm without unlabeled data. When *m* becomes large, the red curve increases dramatically. However, when we add 300 or 600 unlabeled data, the error curves begin to increase very slowly. This coincides with our theory that using unlabeled data can enlarge the range of *m* in the distributed method.

This paper studied the convergence rate of the distribute gradient descent MEE algorithm in a semi-supervised setting. Our results demonstrated that using additional unlabeled data can improve the learning performance of the distributed MEE algorithm, especially in enlarging the range of *m* to guarantee the learning rate. As we know, there are many gaps between theory and empirical studies. We regard this paper as mainly a theoretical paper and expect that the theoretical analysis give some guidance to real applications.

## Figures and Tables

**Table 1:** List of notations used throughout the paper.

Notation	Meaning of the Notation
*X*	the explanatory variable
*Y*	the response variable
X	X∈X, a compact subset of an Euclidian space $R^{n}$
Y	Y∈Y, a subset of R
ρ(·,·)	a Boreal measure on X×Y
$\rho_{X}$	the marginal probability measure of ρ on X
ρ(y|x)	the conditional probability measure of y∈Y given X=x
$g_{\rho }\left(x\right)$	the mean regression function $g_{\rho }\left(x\right)=\int_{Y}yd\rho \left(y|x\right)$
$f_{\rho }\left(x,u\right)$	the target function of MEE induced by $f_{\rho }\left(x,u\right)=g_{\rho }\left(x\right)-g_{\rho }\left(u\right)$
*K*	a reproducing kernel on X×X
*D*	the labeled data set $D=\{\left(x_{1},y_{1}\right),\ldots ,\left(x_{N},y_{N}\right)\}$
*N*	the size of labeled data set *D*
⌈N/4⌉	the largest integer not exceeding N/4
|D|	the cardinality of D, |D|=N
$D^{*}$	the unlabeled data set $D^{*}=\left\{x_{1},\ldots ,x_{S}\right\}$
*S*	the size of unlabeled data set $D^{*}$
$|D^{*}|$	the cardinality of $D^{*},$ $|D^{*}|=S$
$\tilde{D}$	training data set used in the distributed MEE algorithm, consisting of *D* and $D^{*}$
$|\tilde{D}|$	the cardinality of $\tilde{D},$ $|\tilde{D}|=N+S$
*m*	the number of local machines
${\tilde{D}}_{l}$	the *l*th subset of $\tilde{D},$ 1≤l≤m
*G*	the loss function of MEE algorithm
$L_{K}$	the integral operator associated with *K*
$L_{K,\tilde{D}}$	the empirical operator of $L_{K}$ on $\tilde{D}$
$f_{t+1,D}$	the function output by the kernel gradient descent MEE algorithm
	with data *D* and kernel *K* after *t* iterations
$f_{t+1,D_{l}}$	the function output by the kernel gradient MEE algorithm
	with data $D_{l}$ and kernel *K* after *t* iterations
${\bar{f}}_{t+1,\tilde{D}}$	the global output averaging over local outputs $f_{t+1,{\tilde{D}}_{l}},l=1,\ldots ,m$

## Appendix A. Proof of Lemma 3

We state two useful lemmas as follows, whose proof can be found in Reference [22].

**Lemma** **A1.**
*For λ>0, 0<η<1 and j=1,⋯,t-1, we have:*

$$\begin{array}{c} \max\{∥\eta \left(L_{K}+\lambda \right)\pi_{j+1}^{t}\left(L_{K}\right)∥,∥\eta \left(L_{K,\tilde{D}}+\lambda \right)\pi_{j+1}^{t}\left(L_{K,\tilde{D}}\right)∥\}\leq \frac{1}{t-j}+\eta \lambda ,\\ \max\{∥\sum_{j=1}^{t}\eta \left(L_{K}+\lambda \right)\pi_{j+1}^{t}\left(L_{K}\right)∥,∥\sum_{j=1}^{t}\eta \left(L_{K,\tilde{D}}+\lambda \right)\pi_{j+1}^{t}\left(L_{K,\tilde{D}}\right)∥\}\leq 1+\eta \lambda t. \end{array}$$

**Lemma** **A2.**
*For any λ>0 and $f^{*}\in H_{K},$ there holds:*

(A1) $$\begin{array}{c} ∥f_{t+1,\tilde{D}}-f_{t+1}{∥}_{L^{2}}\leq B_{\tilde{D},\lambda }C_{\tilde{D},\lambda }\sum_{i=1}^{t}\left[{\left(t-i\right)}^{-1}+\eta \lambda \right]{∥f_{i}-f^{*}∥}_{K}\\ +B_{\tilde{D},\lambda }\left(1+\eta \lambda t\right)\left(C_{\tilde{D},\lambda }{∥f^{*}∥}_{K}+G_{\tilde{D},\lambda }\right)+c_{p,M}{\left(N+S\right)}^{2p+1}N^{-(2p+1)}t^{p+1/2}h^{-2p}, \end{array}$$

*and:*

(A2) $$\begin{array}{c} ∥f_{t+1,\tilde{D}}-f_{t+1}{∥}_{K}\leq B_{\tilde{D},\lambda }C_{\tilde{D},\lambda }\sum_{i=1}^{t}\left[{\left(t-i\right)}^{-1}+\eta \lambda \right]{∥f_{i}-f^{*}∥}_{K}/\sqrt{\lambda }\\ +B_{\tilde{D},\lambda }\left(1+\eta \lambda t\right)\left(C_{\tilde{D},\lambda }{∥f^{*}∥}_{K}+G_{\tilde{D},\lambda }\right)/\sqrt{\lambda }+c_{p,M}{\left(N+S\right)}^{2p+1}N^{-(2p+1)}t^{p+1/2}h^{-2p}, \end{array}$$

*where the constant $c_{p,M}=2^{4p+2}c_{p}C_{G}^{2p+1}M^{2p+1}.$*

**Proof** **of** **Lemma** **A2.** By Equations (10) and (13), we get a two error decomposition for $f_{t+1,D}-f_{t+1}.$ The first one is:

(A3) $$\begin{array}{c} f_{t+1,\tilde{D}}-f_{t+1}=\eta \sum_{i=1}^{t}\pi_{i+1}^{t}\left(L_{K,\tilde{D}}\right)\left[L_{K}-L_{K,\tilde{D}}\right]\left(f_{i}\right)\\ +\eta \sum_{i=1}^{t}\pi_{i+1}^{t}\left(L_{K,\tilde{D}}\right)\left[f_{\rho ,\tilde{D}}-L_{K}\left(f_{\rho }\right)\right]+\eta \sum_{i=1}^{t}\pi_{i+1}^{t}\left(L_{K,\tilde{D}}\right)E_{i,\tilde{D}}, \end{array}$$

and the second one is:

(A4) $$\begin{array}{c} f_{t+1,\tilde{D}}-f_{t+1}=\eta \sum_{i=1}^{t}\pi_{i+1}^{t}\left(L_{K}\right)\left[L_{K}-L_{K,\tilde{D}}\right]\left(f_{i,\tilde{D}}\right)\\ +\eta \sum_{i=1}^{t}\pi_{i+1}^{t}\left(L_{K}\right)\left[f_{\rho ,\tilde{D}}-L_{K}\left(f_{\rho }\right)\right]+\eta \sum_{i=1}^{t}\pi_{i+1}^{t}\left(L_{K}\right)E_{i,\tilde{D}}. \end{array}$$

It has been proven in Reference [22] that $\left\{f_{t,\tilde{D}}\right\}$ as $∥f_{t,\tilde{D}}{∥}_{K}\leq 2C_{G}\frac{|\tilde{D}|}{\left|D\right|}Mt^{\frac{1}{2}}=2C_{G}\frac{\left|N+S\right|}{N}Mt^{\frac{1}{2}}.$ It follows from Equation (4) that:

(A5) $$\begin{array}{c} ∥E_{t,\tilde{D}}{∥}_{K}\leq \frac{1}{{\left|N+S\right|}^{2}}\sum_{\begin{array}{c} \left(x_{i},y_{i}\right)\in \tilde{D},\\ \left(x_{j},y_{j}\right)\in \tilde{D} \end{array}}∥\left[G^{\prime }\left(\frac{{\left(f_{t,D^{*}}\left(x_{i},x_{j}\right)-y_{i}+y_{j}\right)}^{2}}{h^{2}}\right)-G^{\prime }\left(0\right)\right]\left(f_{t,\tilde{D}}\left(x_{i},x_{j}\right)-y_{i}+y_{j}\right)K_{\left(x_{i},x_{j}\right)}{∥}_{K}\\ \leq c_{p}\frac{{\left(2\frac{\left|N+S\right|}{N}C_{G}M+2\right)}^{2p+1}}{h^{2p}}{∥f_{t,\tilde{D}}∥}_{K}^{2p+1}\leq 2^{4p+2}c_{p}C_{G}^{2p+1}M^{2p+1}\frac{{\left|N+S\right|}^{2p+1}}{N^{2p+1}}t^{p+1/2}h^{-2p}. \end{array}$$

Then, we can follow the proof procedure in Proposition 1 of Reference [24] to prove Equations (A2) and (A1). □

With the help of the lemmas above, we can prove Lemma 3.

**Proof** **of** **Lemma** **3.** Applying Equation (24) with $\tilde{D}={\tilde{D}}_{l}$ for l=1,⋯,m, we have that:

$$\begin{array}{c} ∥{\bar{f}}_{T+1,{\tilde{D}}_{l}}-f_{T+1}{∥}_{L^{2}}={∥\frac{1}{m}\sum_{l=1}^{m}\left({\bar{f}}_{T+1,{\tilde{D}}_{l}}-f_{T+1}\right)∥}_{L^{2}}\\ \leq ∥\eta \sum_{i=1}^{T}\pi_{i+1}^{T}\left(L_{K}\right)\frac{1}{m}\sum_{l=1}^{m}\left[L_{K}-L_{K,{\tilde{D}}_{l}}\right]\left(f_{i,{\tilde{D}}_{l}}\right){∥}_{L^{2}}\\ +∥\eta \sum_{i=1}^{T}\pi_{i+1}^{T}\left(L_{K}\right)\frac{1}{m}\sum_{l=1}^{m}\left[{\hat{f}}_{\rho ,{\tilde{D}}_{l}}-L_{K}\left(f_{\rho }\right)\right]{∥}_{L^{2}}+{∥\eta \sum_{i=1}^{T}\pi_{i+1}^{T}\left(L_{K}\right)\frac{1}{m}\sum_{l=1}^{m}E_{i,{\tilde{D}}_{l}}∥}_{L^{2}}\\ :=I_{1}+I_{2}+I_{3}. \end{array}$$

Firstly, we will bound $I_{1},$ which is most difficult to handle. It can be decomposed as:

$$\begin{array}{cc} I_{1} & \leq {∥\eta \sum_{i=1}^{T}\pi_{i+1}^{T}\left(L_{K}\right)\frac{1}{m}\sum_{l=1}^{m}{\left(L_{K}+\lambda \right)}^{\frac{1}{2}}\left[L_{K}-L_{K,{\tilde{D}}_{l}}\right]\left(f_{i,{\tilde{D}}_{l}}-f_{i}\right)∥}_{K}\\ & +{∥\eta \sum_{i=1}^{T}\pi_{i+1}^{T}\left(L_{K}\right)\frac{1}{m}\sum_{l=1}^{m}{\left(L_{K}+\lambda \right)}^{\frac{1}{2}}\left[L_{K}-L_{K,{\tilde{D}}_{l}}\right]\left(f_{i}-f^{*}\right)∥}_{K}\\ & +{∥\eta \sum_{i=1}^{T}\pi_{i+1}^{T}\left(L_{K}\right)\frac{1}{m}\sum_{l=1}^{m}{\left(L_{K}+\lambda \right)}^{\frac{1}{2}}\left[L_{K}-L_{K,{\tilde{D}}_{l}}\right]\left(f^{*}\right)∥}_{K}\\ & :=I_{11}+I_{12}+I_{13}. \end{array}$$

Then, it is easy to get that by Lemma A1 and $f_{1,{\tilde{D}}_{l}}=f_{1}=0$:

$$\begin{array}{cc} I_{11} & ={∥\sum_{i=1}^{T}\eta \left(L_{K}+\lambda \right)\pi_{i+1}^{T}\left(L_{K}\right)\frac{1}{m}\sum_{l=1}^{m}{\left(L_{K}+\lambda \right)}^{-\frac{1}{2}}\left[L_{K}-L_{K,{\tilde{D}}_{l}}\right]\left(f_{i,{\tilde{D}}_{l}}-f_{i}\right)∥}_{K}\\ & \leq \sum_{i=1}^{T}∥\eta \left(L_{K}+\lambda \right)\pi_{i+1}^{T}\left(L_{K}\right)∥{∥\frac{1}{m}\sum_{l=1}^{m}{\left(L_{K}+\lambda \right)}^{-\frac{1}{2}}\left[L_{K}-L_{K,{\tilde{D}}_{l}}\right]\left(f_{i,{\tilde{D}}_{l}}-f_{i}\right)∥}_{K}\\ & \leq \sum_{i=1}^{T}\left(\eta \lambda +{\left(T-i\right)}^{-1}\right)\underset{1\leq l\leq m}{\sup}{∥{\left(L_{K}+\lambda \right)}^{-\frac{1}{2}}\left[L_{K}-L_{K,{\tilde{D}}_{l}}\right]\left(f_{i,{\tilde{D}}_{l}}-f_{i}\right)∥}_{K}\\ & \leq \underset{1\leq l\leq m}{\sup}\sum_{i=1}^{T}\left(\eta \lambda +{\left(T-i\right)}^{-1}\right){∥f_{i,{\tilde{D}}_{l}}-f_{i}∥}_{K}C_{{\tilde{D}}_{l},\lambda }. \end{array}$$

Applying Proposition A2 with $\tilde{D}={\tilde{D}}_{l}$ and t+1=i, we have that:

$$\begin{array}{c} ∥f_{i,{\tilde{D}}_{l}}-f_{i}{∥}_{K}\leq \sum_{s=1}^{i-1}\left({\left(i-s-1\right)}^{-1}+\lambda \eta \right){∥f_{s}-f^{*}∥}_{K}B_{{\tilde{D}}_{l},\lambda }C_{{\tilde{D}}_{l},\lambda }\lambda^{-\frac{1}{2}}\\ +\left(1+\lambda \eta i\right)B_{{\tilde{D}}_{l},\lambda }(C_{{\tilde{D}}_{l},\lambda }∥f^{*}{∥}_{K}+G_{{\tilde{D}}_{l},\lambda })\lambda^{-\frac{1}{2}}+c_{p,M}\frac{{\left|N+S\right|}^{2p+1}}{N^{2p+1}}i^{p+1/2}h^{-2p}. \end{array}$$

Thus:

(A6) $$\begin{array}{cc} I_{11} & \leq \underset{1\leq l\leq m}{\sup}\sum_{i=1}^{T}\left(\eta \lambda +{\left(T-i\right)}^{-1}\right)C_{{\tilde{D}}_{l},\lambda }\times \{\sum_{s=1}^{i-1}\left({\left(i-s-1\right)}^{-1}+\lambda \eta \right){∥f_{s}-f^{*}∥}_{K}B_{{\tilde{D}}_{l},\lambda }C_{{\tilde{D}}_{l},\lambda }\lambda^{-\frac{1}{2}}\\ & +\left(1+\lambda \eta i\right)B_{{\tilde{D}}_{l},\lambda }(C_{{\tilde{D}}_{l},\lambda }∥f^{*}{∥}_{K}+G_{{\tilde{D}}_{l},\lambda })\lambda^{-\frac{1}{2}}+c_{p,M}\frac{{\left|N+S\right|}^{2p+1}}{N^{2p+1}}i^{p+1/2}h^{-2p}\}. \end{array}$$

By Lemma A1 again, we have:

(A7) $$\begin{array}{cc} I_{12} & \leq {∥\sum_{i=1}^{T}\eta \left(L_{K}+\lambda \right)\pi_{i+1}^{T}\left(L_{K}\right)\frac{1}{m}\sum_{l=1}^{m}{\left(L_{K}+\lambda \right)}^{-\frac{1}{2}}\left[L_{K}-L_{K,{\tilde{D}}_{l}}\right]\left(f_{i}-f^{*}\right)∥}_{K}\\ & \leq \sum_{i=1}^{T}∥\eta \left(L_{K}+\lambda \right)\pi_{i+1}^{T}\left(L_{K}\right)∥{∥\frac{1}{m}\sum_{l=1}^{m}{\left(L_{K}+\lambda \right)}^{-\frac{1}{2}}\left[L_{K}-L_{K,{\tilde{D}}_{l}}\right]∥∥f_{i}-f^{*}∥}_{K}\\ & \leq \sum_{i=1}^{T}\left(\eta \lambda +{\left(T-i\right)}^{-1}\right)D_{\tilde{D},\lambda }{∥f_{i}-f^{*}∥}_{K}, \end{array}$$

and:

(A8) $$\begin{array}{cc} I_{13} & \leq ∥\sum_{i=1}^{T}\eta \left(L_{K}+\lambda \right)\pi_{i+1}^{T}\left(L_{K}\right)∥∥\frac{1}{m}\sum_{l=1}^{m}{\left(L_{K}+\lambda \right)}^{-\frac{1}{2}}\left[L_{K}-L_{K,{\tilde{D}}_{l}}\right]∥{∥f^{*}∥}_{K}\\ & \leq \left(1+\lambda \eta T\right)D_{\tilde{D},\lambda }{∥f^{*}∥}_{K}. \end{array}$$

This completes the estimate of $I_{1}$ with Equations (A6), (A7), and (A8). Now we turn to bound $I_{2}.$ Then, by the definition of $F_{\tilde{D},\lambda }$, the bound (Equation (A5)) of $E_{t,\tilde{D}}$ and Lemma A1, we obtain that:

$$\begin{array}{c} I_{2}\leq \left(1+\eta \lambda T\right)F_{\tilde{D},\lambda }, \end{array}$$

and:

$$\begin{array}{c} I_{3}\leq c_{p,M}\frac{|\tilde{D}{|}^{2p+1}}{{\left|D\right|}^{2p+1}}T^{p+3/2}h^{-2p}=c_{p,M}\frac{{\left|N+S\right|}^{2p+1}}{N^{2p+1}}T^{p+3/2}h^{-2p}. \end{array}$$

Together with the bound of Equations (A6), (A7), and (A8), we can get the desired conclusion (Equation (15)). □
